# Circular RNA 0001789 sponges miR-140-3p and regulates PAK2 to promote the progression of gastric cancer

**DOI:** 10.1186/s12967-022-03853-2

**Published:** 2023-02-05

**Authors:** Jun You, Yinan Chen, Donghan Chen, Yongwen Li, Tinghao Wang, Jingtao Zhu, Qingqi Hong, Qiyuan Li

**Affiliations:** 1 Department of Gastrointestinal Oncology Surgery, Cancer Center, The First Affiliated Hospital of Xiamen University, School of Medicine, Xiamen University, Xiamen, Fujian 361001 China; 2 The Third Clinical Medical College, Fujian Medical University, Xiamen, Fujian 361001 China; 3 National Institute of Data Science in Health and Medicine, School of Medicine, Xiamen University, Xiamen, Fujian 361100 China

**Keywords:** Gastric cancer, Circular RNA 0001789, MicroRNA-140-3p, p21 activated kinase 2, Epithelial-mesenchymal transition

## Abstract

**Background:**

Gastric cancer (GC) is the third-leading cause of cancer-associated mortalities globally. The deregulation of circular RNAs (circRNAs) and microRNAs (miRNAs or miRs) is widely implicated in the pathogenesis and progression of different cancer types.

**Methods:**

The expression profiling of circRNAs in GC is required to identify crucial circRNAs as biomarkers or therapeutic targets. In the present study, a published circRNA microarray dataset was used to identify differentially expressed circRNAs between GC tissues and normal gastric mucosa tissues. Reverse transcription-quantitative PCR was performed to validate the expression of circ_0001789. Fisher’s exact test, receiver operating characteristic curve and Kaplan-Meier plots were employed to analyze the clinical significance of circ_0001789. The miRNA targets of circ_0001789 were predicted using an online database, and their functional interaction was further confirmed by RNA pull-down, RNA immunoprecipitation and dual luciferase reporter assays. Transwell assays were conducted to investigate the biological functions of circ_0001789, miR-140-3p and p21 activated kinase 2 (PAK2) in the migration and invasion of GC cells. A xenograft mouse model was established to validate the role of circ_0001789 in the tumorigenesis of GC cells.

**Results:**

circ_0001789 was identified as a highly expressed circRNA in GC tissues versus normal gastric mucosa tissues. Silencing circ_0001789 attenuated the malignancy of GC cells, and exosomal circ_0001789 was sufficient to regulate the malignant phenotype of GC cells. miR-140-3p was further identified as a downstream target of circ_0001789, which showed a negative correlation with circ_0001789 expression in GC tissues. Overexpression of miR-140-3p suppressed cell migration, invasion and epithelial-mesenchymal transition in GC cells. PAK2 was identified as the target of miR-140-3 to mediate the malignant phenotype of GC cells.

**Conclusion:**

The present data suggested that the upregulation of circ_0001789 was associated with the progression of GC and with poor prognosis in patients with GC, and that miR-140-3p/PAK2 served as the downstream axis to mediate the oncogenic effect of circ_0001789.

**Supplementary Information:**

The online version contains supplementary material available at 10.1186/s12967-022-03853-2.

## Introduction

Gastric cancer (GC) remains a global health challenge, as it is the third-leading cause of cancer-associated mortalities [1, 2]. Notably, the treatment of early diagnosed GC can lead to a favorable outcome. However, the majority of GC cases are diagnosed at advanced stage, and such patients miss the opportunity for optimal treatment. In this regard, a comprehensive understanding of genetic and other type of biomarkers in the early stage of GC could facilitate early diagnosis, and the elucidation of the mechanisms underlying the progression of GC could provide insights into the development of novel therapies.

Non-coding RNAs have been implicated in the initiation and development of GC. Circular RNAs (circRNAs) are a class of stable non-coding RNAs that are resistant to ribonuclease R digestion due to their covalently closed loop structure [3]. Due to their stability, circRNAs are not only present in their corresponding source tissues, but are also abundant in blood, and have been proposed as promising biomarkers in different tumors [4, 5]. Recently, tumor-associated circRNAs have been regarded as potential biomarkers for GC diagnosis and progression [6–8].

Exosomes are extracellular vesicles secreted by cells, which carry cellular information and products to modulate cell–cell communication [9]. The roles of exosomes produced by GC and their components are worthy of investigation, as they may serve as potential GC biomarkers [2, 9]. A previous report showed that exosomal circRNAs could indicate the malignant characteristics of GC [10]. Recent technological advancements and the availability of information on deregulated circRNAs in cancer databases had also shed light on the different roles of circRNAs in various cancer types. In addition to circRNAs, microRNAs (miRNAs or miRs) are another class of short non-coding RNAs that are involved in the progression of GC [11, 12]. For example, miR-140-3p has been reported as a tumor suppressor that inhibits the metastasis of various types of cancer [13, 14]. miR-140-3p could also promote autophagy, and alleviate inflammatory and oxidative stress to mitigate cell damage [15].

A member of the serine/threonine kinase p21 activated kinase (PAK) family, PAK2, has become a promising anticancer target that regulates the proliferation and survival of GC cells [16–18]. PAK2 could act as a downstream substrate of the Rho family of GTPases to regulate the cytoskeleton and cell migration [19]. However, the functional interactions between miR-140-3p and its associated circRNAs or downstream target genes in GC remain elusive. The current study analyzed the expression profile of circRNAs in GC, and demonstrated the role of exosome-derived circ_0001789 in the progression of GC. The biological functions of circ_0001789 in GC were investigated by gain and loss of function studies. Furthermore, the present study investigated the molecular mechanisms by which circ_0001789 interacts with the miR-140-3p/PAK2 axis to modulate the progression of GC.

## Materials and methods

### Clinical samples

Normal gastric mucosa tissue samples of healthy controls (n = 50) and gastric mucosa tissues of patients with GC (n = 70) were collected at The First Affiliated Hospital of Xiamen University (Xiamen, China). The controls were healthy individuals with matched ages and sex to those of the patients with GC. Patients who underwent chemotherapy or radiotherapy were excluded from the study. All the enrolled participants had provided informed consent. The study protocols were approved by the Research Ethics Committee of The First Affiliated Hospital of Xiamen University, and the study was carried out following the Declaration of Helsinki.

### circRNA microarray data

To identify the differentially expressed circRNAs between GC tissues and normal gastric mucosa tissues, a published circRNA microarray dataset (GSE83521) was extracted, which contained 50 gastric mucosa samples from healthy controls and 70 samples from patients with GC. Cufflinks v2.2.1 was used to derive expression (fragments per kilobase million) values, and differential gene expression was performed with the Cuffdiff package. False Discovery Rate (FDR)-adjusted P-value following Benjamini-Hochberg correction for multiple comparisons was used to define differential expression. CircRNAs with FDR-adjusted P < 0.05 and log2 fold-change  > 1 were considered as significantly differentially expressed.

### Reagents and cells

All cell lines were purchased from American Type Culture Collection. Primary antibodies against E-cadherin, N-cadherin, vimentin, VEGF-A, PAK2, Ki67, CD63, tumor susceptibility gene 101, calnexin and CD34 were purchased from Abcam. A chemiluminescence imaging kit was obtained from Beyotime Institute of Biotechnology. RPMI-1640, Ham’s F-12 K and MEM media, as well as fetal bovine serum (FBS) were obtained from Gibco (Thermo Fisher Scientific, Inc.). Short hairpin RNA (shRNA) (sh-circ#1, sh-circ#2 and sh-circ#3) and sh-negative control (NC), as well as pcDNA-PAK2, vector-exo, circ_0001789-exo, empty vector (vector), miRNA controls (miR-NC), and miR-430-3p mimic or inhibitor (miR-430-3p or anti-miR-430-3p) were purchased from Shanghai GeneChem Co., Ltd. Lipofectamine™ 2000 transfection reagent was obtained from Thermo Fisher Scientific, Inc. Goat anti-rabbit and mouse horseradish peroxidase (HRP)-conjugated IgG antibodies were purchased from Santa Cruz Biotechnology, Inc. 3-Methyladenine was purchased from Sigma-Aldrich (Merck KGaA). Pierce™ Magnetic RNA-Protein Pull-Down Kit was obtained from Thermo Fisher Scientific, Inc. Primers were synthesized by Integrated DNA Technologies, Inc.

### Cell culture, plasmid vector, group design and transfection

GC cell lines (AGS, HGC27, MKN-45 and MKN-74), primary human umbilical vein endothelial cells (HUVECs) and the human gastric mucosal epithelial cell line (GES-1) were maintained in RPMI-1640, Ham’s F-12 K or MEM media (Gibco; Thermo Fisher Scientific, Inc.) supplemented with 10% FBS, penicillin/streptomycin (1:100; Sigma-Aldrich; Merck KGaA), 4 mM l-glutamine and 0.19% HEPES (Sigma-Aldrich; Merck KGaA) in a 37 °C incubator with 5% CO_2_. The usage of primary human cells in the present study was approved by the Research Ethics Committee at The First Affiliated Hospital of Xiamen University.

The shRNA sequences (sh-circ#1, sh-circ#2 and sh-circ#3) were synthesized by Shanghai GeneChem Co., Ltd., and the sequences are summarized in Additional file 2: Table S1. The shRNA sequences were cloned into the pLKO.1-Puro vector for shRNA-mediated gene silencing. To overexpress circ_0001789, the sequence of circ_0001789 was synthesized by Integrated DNA Technologies, Inc. and cloned into the pLCDH-ciR vector (Guangzhou RiboBio Co., Ltd.). The transfection of of circ_0001789 shRNA or expression vector was conducted as follows [20]: Initially, cells were seeded in a 6-well plate for 24 h and allowed to reach 60% confluence. The PLUS^™^ reagent of the Lipofectamine^™^ 2000 transfection kit was mixed with 3 μg vector in 150 μl serum-free medium at room temperature. Next, the mixtures were combined with an equal volume of serum-free medium containing 6 μl Lipofectamine^™^ 2000. After 30 min incubation, the transfection solution was added to the cells, and complete medium with FBS was added 12 h after transfection for an additional 48 h culture before experimental analysis.

### Cell proliferation assay

The proliferation of the GC cells subjected to different treatments was measured by Cell Counting Kit (CCK)-8 assay. Briefly, GC cells were seeded in 96-well plates at a density of 1 × 10^4^ cells per well, and cultured for 24, 48 and 72 h. CCK-8 solution (1:50 dilution) was added into the cell culture at the indicated time points and incubated for 3 h at 37 °C. Finally, the absorbance at 450 nm was determined with a EnSpire Multi-label Plate Reader (PerkinElmer, Inc.).

### RNA pull-down assay

Cells (0.25 × 10^5^) seeded in 6-well plates were transfected with biotinylated control or circRNA probe (100 nM) using Lipofectamine™ 2000. After 48 h, cells lysates (from 1 × 10^6^ cells) were collected by using IP Lysis Buffer (Beyotime Biotechnology), and 10% of the lysates was employed as the input. The remaining lysates were incubated with M-280 streptavidin magnetic beads (Sigma-Aldrich) at 4 °C overnight. A magnetic bar was used to precipitate the magnetic beads. After 4 washes with a high-salt wash buffer, both the input and the precipitated samples from the pull-down were purified with TRIzol^®^ reagent (Invitrogen). The enriched miRNA was then detected by reverse transcription-quantitative PCR (RT-qPCR) analysis.

### Dual luciferase reporter assay

The sequence containing the wild-type (WT) binding site or the mutated (MUT) binding site was cloned into the firefly luciferase PmirGLO reporter vector (Promega Corporation). The reporter plasmid and *Renilla* luciferase (hRlucneo) control plasmid were co-transfected into cells in the presence of miRNA mimic or miR-NC in a 12-well plate using Lipofectamine^™^ 2000 reagent. At 48 h post-transfection, the relative luciferase activities were measured using Luciferase Reporter Gene Assay System (PerkinElmer, Inc.) on GloMax^®^ Discover Microplate Reader (Promega Corporation).

### RNA immunoprecipitation (RIP) assay

Cell lysates (from 1 × 10^6^ cells) collected by 1 mL IP lysis buffer were incubated with 100 μl protein-A magnetic beads (Sigma) coated with 10 μg control IgG or anti-AGO2 antibody at 4 °C overnight. The magnetic beads were precipitated using a magnetic bar, and the precipitated samples were washed 3 times with washing buffer. The eluted samples were purified with TRIzol^®^ reagent (Invitrogen; Thermo Fisher Scientific, Inc.). The relative level of precipitated molecules was detected using RT-qPCR analysis and normalized to that in the input samples.

### Western blotting

A total amount of 10 µg protein sample was subjected to 10 or 12% SDS-PAGE, followed by the transfer to PVDF membranes (MilliporeSigma). After blocking with 5% skimmed milk, the membrane was incubated with primary antibodies against E-cadherin, N-cadherin, vimentin VEGF-A and actin (1:1000, all from Abcam) at 4 °C. After further probing with HRP-linked secondary antibodies at room temperature for 1 h, the protein bands were visualized using an enhanced chemiluminescence kit (Santa Cruz Biotechnology, Inc.) and photographed on a gel imaging system (Bio-Rad Laboratories, Inc.). Densitometry analysis was performed with Image J software [21].

### Mouse xenografts

The animal experiments were approved by the Institutional Animal Care and Use Committee of the First Affiliated Hospital of Xiamen University (Xiamen, China). NU/NU mice (6 weeks old) were obtained from the Experimental Animal Center of Xiamen University. For establishing the xenograft model, 1 × 10^6^ AGS cells transfected with sh-NC and sh-circ-0001789 were injected subcutaneously on the back of the mice. Tumor growth was monitored using a Vernier caliper every 7 days, and tumor volume (V) was calculated as V = length x (width)^2^/2 [22]. If the tumor xenograft exceeded 2,000 mm^3^, the mice would be euthanized immediately. For euthanasia, a chamber was connected to a carbon dioxide cylinder, and the flow rate was adjusted to displace 40% of the cage volume per min. Mice were placed into the euthanizing chamber for 10 min until no movement was observed. Animal death was confirmed by subsequent cervical dislocation.

### Immunohistochemistry (IHC) staining

Paraffin-embedded tissues were cut into 4-5 μm sections using a microtome. After de-paraffinization and hydration, antigen retrieval was conducted by boiling the slides in citrate buffer for 90 s, followed by the incubation with 3% hydrogen peroxide for 10 min. After 3 washes in TBST buffer, the sections were blocked for 1 h in TBST with 5% normal goat serum, and then probed with primary antibodies (1:500) overnight at 4 °C. Following washing with TBST buffer, the sections was soaked with 1–3 drops of SignalStain® Boost Detection Reagent (Cell Signaling Technologies, Inc.) and incubated in a humidified chamber for 30 min at room temperature. The signal was developed using 400 μl SignalStain® substrate (Cell Signaling Technologies, Inc.) for 5 min. The sections were mounted with coverslip using mounting medium (Cell Signaling Technologies, Inc.) before being imaged under a bright-field microscope [23, 24].

### Cell migration and invasion assays

Transfected cells were collected after 48 h and re-suspended in serum-free DMEM. The Transwell inserts were pre-coated with 1% Matrigel at 37 °C for 1 h. Approximately 5 × 10^5^ cells were inoculated into the upper chamber, while 500 μl 10% serum-containing medium was added to the lower chamber. After 18 h, cells on the membrane were fixed with 4% paraformaldehyde for 10 min and stained with 0.5% crystal violet (Sigma-Aldrich) for 20 min. Cells were photographed under a microscope (Olympus Corporation). Transwell migration assay was performed in Transwell inserts without Matrigel coating, and the other procedures were the same as those described above for the invasion assay.

### RT-qPCR analysis

Total RNA from GC tissues and cells was extracted using RNeasy Mini Kit (Qiagen GmbH) or mirVana miRNA Isolation Kit (Thermo Fisher Scientific, Inc.). Exosomes were purified from serum samples or cell cultures using Total Exosome Isolation Kit (Invitrogen; Thermo Fisher Scientific, Inc.), and total RNA was purified from exosome samples using TRIzol^®^ reagent (Invitrogen; Thermo Fisher Scientific, Inc). Total RNA (5 μg) was used for RT into cDNA using PrimeScript^™^ RT Master Mix (Takara Bio, Inc.). qPCR analysis was performed in triplicate using Tiangen Master Mix SYBR Green RT-PCR SuperMix (Tiangen Biotech Co., Ltd.) on a CFX96^™^ Real-Time PCR Detection System (Bio-Rad Laboratories, Inc.). Actin and U6 small nuclear RNA were used as internal references to normalize mRNA and miRNA, respectively. The sequences of the primers used in the present study are summarized in Additional file 3: Table S2.

### RNase R and actinomycin D treatment

RNase R (Takara Bio, Inc.) was applied to digest the RNA samples in order to evaluate their stability. Total RNA samples were divided equally into two portions: One was digested with RNase R (RNase R + group), while the other one was used as a control (mock group). The two samples were incubated at 37 °C for 25 min. The relative quantity of linear RAB11 family interacting protein 1 (RAB11FIP1) mRNA and circ_0001789 in each sample was detected by RT-qPCR.

For the RNA stability assay, transcription was blocked by 3 μg/ml actinomycin D (Sigma-Aldrich; Merck KGaA) for the indicated duration, and RNA samples were collected with TRIzol®. The stability of circ_0001789 and RAB11FIP1 mRNA was analyzed by RT-qPCR by comparison with the samples before treatment (0 h time point).

### Endothelial cell tube formation assay

An in vitro angiogenesis assay kit (Abcam) was used to evaluate angiogenic potential. In brief, 40 μl extracellular matrix solution was added to a pre-chilled 96-well culture plate and incubated at 37 °C for 20 min. HUVECs (2.0 × 10^4^) were seeded in a coated plate and incubated with exosomes from different cell lines for 48 h. Endothelial cell tube formation was observed with an inverted microscope in a bright field [25]. Image J Angiogenesis Analyzer (National Institutes of Health) was used for quantification of the network structure.

### *Fluorescence *in situ* hybridization (FISH)*

RNAScope kit (Invitrogen; Thermo Fisher Scientific, Inc.) was used to perform FISH according to the manufacturer’s instructions. Briefly, cells cultured on a coverslip were fixed with 4% paraformaldehyde and permeabilized with 0.05% Triton-X100 for 15 min. Next, 50 nM circ_0001789 probe with cyanine 3 fluorescent dye (Guangzhou RiboBio Co., Ltd.) in hybridization buffer was applied for 3 h incubation at 50 °C. Upon washing with TBST buffer, mounting medium containing DAPI (Vector Laboratories, Inc.) was used to mount the cell samples on the slides. The expression intensity and localization of circ_0001789 was observed with a fluorescence microscope.

### Hematoxylin and eosin (H&E) staining

H&E staining was performed in lung tissues using H&E Stain Kit (Abcam). Deparaffinized/hydrated sections were incubated in a sufficient volume of hematoxylin solution (Mayer’s) to completely cover the tissue section for 5 min. The section was then rinsed twice with distilled water and incubated with Bluing Reagent for 60 s. Upon washing, the section was dehydrated in absolute alcohol and stained with eosin Y-solution for 3 min. The sections were rinsed in absolute ethanol 3 times, and images were collected under an inverted microscope.

### Statistical analysis

Data are expressed as the mean ± SD, and were analyzed with GraphPad Prism 9.0 (GraphPad Software, Inc.). Statistical significance between two groups was assessed with paired two-tailed Student’s t-test, while comparisons among multiple groups were performed with one-way or two-way ANOVA followed by Tukey’s post hoc test. χ^2^ test was applied to investigate the association between GC and the circ_0001789 expression level. Kaplan Meier Curve and log-rank test were used to compare the cumulative survival rates in patients. ^*^P < 0.05, ^**^P < 0.01, ^***^P < 0.001.

## Results

### Upregulation of circ_0001789 in GC specimens is associated with poor prognosis

**Fig. 1:**
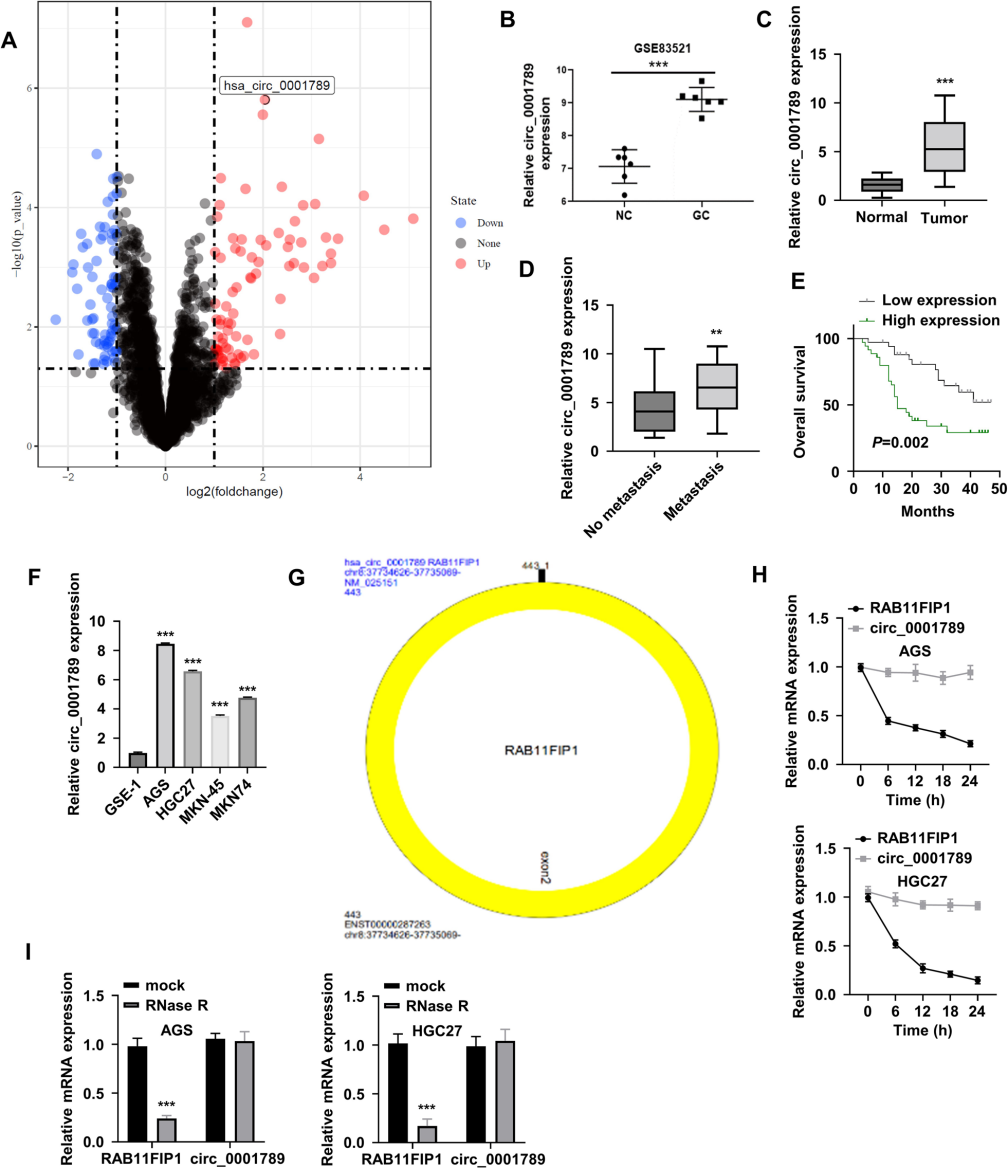
Upregulation of circ_0001789 in GC clinical samples is associated with poor prognosis. **A** Volcano plot showing differentially expressed circRNA expression between GC samples and normal gastric mucosa samples in the GSE83521 dataset. **B** Relative expression level (fragments per kilobase million) of circ_0001789 between normal mucosa tissues (negative control) and GC tissues in the GSE83521 dataset. **C** RT-qPCR analysis of the relative circ_0001789 expression in normal mucosa tissues of healthy controls (n = 50) and GC tumor samples (n = 70). **D** RT-qPCR analysis of the relative circ_0001789 expression in GC samples between patients with and without metastasis. **E** The overall survival rate of patients with GC exhibiting high or low circ_0001789 expression levels was analyzed by Kaplan Meier plotter. **F** Relative expression level of circ_0001789 in GC cell lines (AGS, HGC27, MKN-45 and MKN-74) and in normal gastric epithelial cells (GES-1). **G** Schematic illustration of circ_0001789, which is formed by exon2 of the RAB11FIP1 gene. **H** The relative levels of circ_0001789 and RAB11FIP1 mRNA in AGS and HGC27 cells at different time points (0, 6, 12, 18 and 24 h) were examined by RT-qPCR after actinomycin D treatment. **I** Total RNA was partially digested with RNase R (RNase R^+^ group), while the other part of the sample was used as a control (mock group). The relative level of linear RAB11FIP1 mRNA and circ_0001789 in each sample was detected by RT-qPCR. ^*^P < 0.05, ^**^P < 0.01, ^***^P < 0.001. circ, circular RNA; GC, gastric cancer; RT-qPCR, reverse transcription-quantitative PCR; RAB11FIP1, RAB11 family interacting protein 1

To identify the differentially expressed circRNAs between GC tissues and normal gastric mucosa tissues, a published circRNA microarray dataset (GSE83521) was extracted. Differential expression analysis (log2 fold-change > 1, adjusted P < 0.05) showed that a number of circRNAs were downregulated (as shown in blue) and upregulated (as represented in red) in GC samples (Fig. 1A). Circ_0001789 was identified as one of the most significantly upregulated circRNAs in GC samples (Fig. 1A, B). Since the functional role of circ_0001789 in GC progression is unknown, this circRNA was selected for further investigation. To confirm its expression in GC tumors, gastric mucosa tissue samples were collected from healthy controls (n = 50), while GC tissues were collected from patients with GC (n = 70). RT-qPCR validated that circ_0001789 was highly expressed in GC tissues compared with its expression in normal gastric mucosa samples (Fig. 1C). Compared with that of samples from patients without metastasis, circ_0001789 showed a relatively higher expression level in patients with metastasis (Fig. 1D). To analyze the association of circ_000178 level and patient prognosis, patients with GC were divided into high (n = 35) and low (n = 35) expression groups based on the median expression value of circ_000178 in the 70 patients with GC. The survival rate in patients with GC exhibiting high circ_0001789 expression levels was worse than that of patients with GC and low expression of circ_0001789, as showed by Kaplan-Meier analysis of the survival curve represented in Fig. 1E.

The expression level of circ_0001789 was significantly higher in GC cell lines (AGS, HGC27, MKN-45 and MKN-74) than in normal gastric epithelial cells (GES-1) (Fig. 1F). The association between circ_0001789 level and the clinical features of patients with GC was also examined. As shown in Table 1, a high level of circ_0001789 expression was significantly associated with advanced TNM stage and distal node metastasis. However, there was no significant difference in patient’s age, sex or tumor size between the circ_0001789 low and high-expression groups.

**Table 1:** Correlations of circ_0001789 expression with clinicopathologic features of gastric cancer

Clinicopathological characteristics	Number	Low circ_0001789 expression (n = 35)	High circ_0001789 expression (n = 35)	*P* value
Age				0.1432
≥ 50	42	24	18	
< 50	28	11	17	
Gender				0.4667
Male	41	19	22	
Female	29	16	13	
Tumor size				0.1513
≥ 3 cm	36	15	21	
< 3 cm	34	20	14	
Distant metastasis				0.044
M0	46	27	19	
M1	24	8	16	
TNM stage				0.0168
I + II	36	23	13	
III + IV	34	12	22	

As a circRNA, sequence alignment showed that circ_0001789 was formed by the back splicing of exon2 of RAB11FIP1 (Fig. 1G). To verify its circular structure, transcription was first blocked by actinomycin D, and the RNA decay rate of RAB11FIP1 and circ_0001789 was analyzed. RT-qPCR analysis showed that the RAB11FIP1 mRNA level decreased rapidly following actinomycin D treatment, while circ_0001789 remained relatively stable upon transcription inhibition (Fig. 1H). In addition, RNase R digestion markedly reduced the level of RAB11FIP1 mRNA in the AGS and HGC27 cell lines, whereas it showed little effect on circ_0001789 (Fig. 1I). These data suggested that circ_0001789 possessed a closed-loop structure that was stable and resistant to RNase R degradation.

### *Circ_0001789 participates in cell-to-cell communication *via* exosomes in GC cells*

**Fig. 2:**
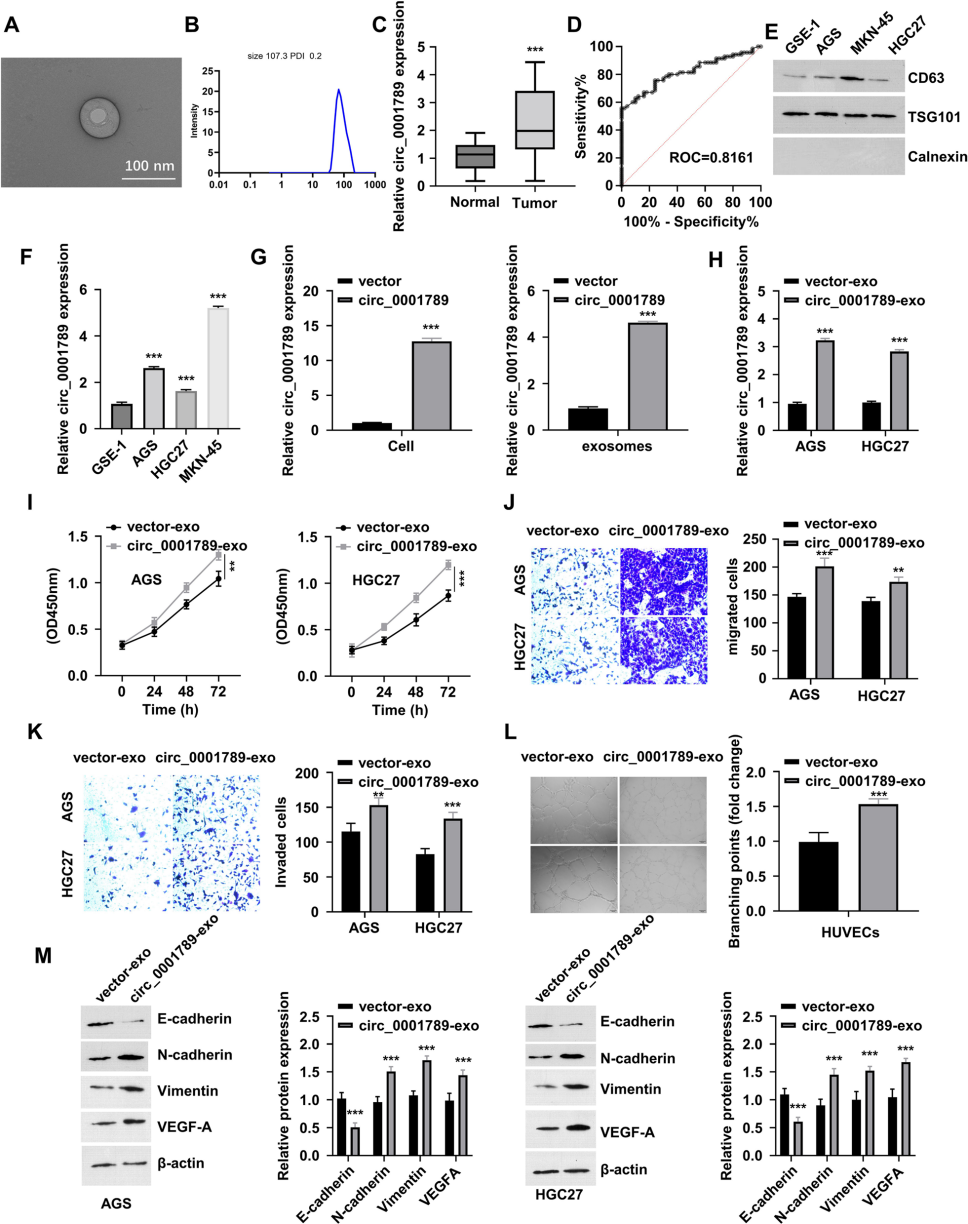
circ_0001789 participates in cell-to-cell communication via exosomes in GC cells. **A** A representative TEM image of exosomes in the serum samples of normal individuals and patients with GC. **B** The diameter of exosomes was analyzed by TEM. **C** Relative circ_0001789 expression level in exosome samples of healthy controls (n = 50) and patients with GC (n = 70). **D** Receiver operating characteristic curve analysis of the specificity and sensitivity of exosomal circ_0001789 as a diagnostic marker for GC. **E** Western blot detection of exosomal markers (CD63 and tumor susceptibility gene 101) in the exosome samples of GC cell lines (AGS, HGC27 and MKN-45) and normal gastric epithelial cells (GES-1). **F** The relative circ_0001789 levels in exosomal samples of GC cell lines (AGS, HGC27 and MKN-45) and normal gastric epithelial cells (GES-1) were detected by RT-qPCR. **G** Relative circ_0001789 expression level in cells and exosomal samples of MKN-45 cells transfected with empty vector or circ_0001789 expression vector. **H** AGS and HGC27 cells were incubated with exosomes from MKN-45 cells transfected with empty vector (vector-exo) or circ_0001789 expression vector (circ_0001789-exo) for 48 h, and the relative circ_0001789 levels were determined by RT-qPCR. **I** Cell Counting Kit-8 proliferation assay in AGS and HGC27 cells treated with vector-exo or circ_0001789-exo. **J** and **K** Cell migration and invasion assays in AGS and HGC27 cells treated with vector-exo or circ_0001789-exo. **L** Tube formation assay in human umbilical vein endothelial cells treated with vector-exo or circ_0001789-exo. **M** Protein levels of E-cadherin, N-cadherin, vimentin and VEGF-A in AGS and HGC27 cells treated with vector-exo or circ_0001789-exo. ^*^P < 0.05, ^**^P < 0.01, ^***^P < 0.001. circ, circular RNA; GC, gastric cancer; TEM, transmission electron microscopy; RT-qPCR, reverse transcription-quantitative PCR

As exosome-derived circRNAs are involved in cell–cell communication, it was hypothesized that circ_0001789 participates in cell-to-cell communication via exosomes. Exosomes were isolated from the serum of normal individuals and patients with GC, and their morphology was examined using transmission electron microscopy (Fig. 2A) and the enumerated diameter (Fig. 2B), which revealed that the exosomes were ~ 100 nm in diameter. RNA was purified the from the exosome samples of healthy controls and patients with GC, and a relatively higher expression of circ_0001789 was observed in the serum samples of patients with GC (Fig. 2C). In addition, the increased expression of exosome circ_0001789 could be a diagnostic indicator, as the area under the curve value of the receiver operating characteristic curve was > 0.8 (Fig. 2D). As shown in Fig. 2E, western blot analysis indicated that the expression level of the exosome marker CD63 was higher in GC cell lines than in GES-1 cells.

Since MKN-45 cells expressed high levels of exosomal markers and exosomal circ_0001789 (Fig. 2E, F), MKN-45 cells were used as donor cells of exosomes, while AGS and HGC27 cells were used as recipient cells. MKN-45 cells were transfected with control and circ_0001789 plasmids, and the circ_0001789 plasmid significantly increased the cellular and exosomal levels of circ_0001789 (Fig. 2G).

Exosomes from MKN-45 cells transfected with control vector (vector-exo) and circ_0001789 plasmid (circ_0001789-exo) were collected and used to treat AGS and HGC27 cells. Circ_0001789-exo incubation significantly increased the cellular level of circ_0001789 in AGS and HGC27 cells (Fig. 2H). In addition, circ_0001789-exo incubation promoted the proliferation of AGS and HGC27 cells, as revealed by the results of CCK-8 proliferation assay (Fig. 2I). Transwell migration and invasion assays were performed, which demonstrated that circ_0001789-exo incubation also enhanced the migratory and invasive capabilities of AGS and HGC27 cells (Fig. 2J, K). Furthermore, tube formation assay in HUVECs suggested that exosome-derived circ_0001789 promoted angiogenesis (Fig. 2L). Changes in epithelial-mesenchymal transition (EMT) biomarkers were also examined, and upregulation of N-cadherin, vimentin and VEGF-A was observed, as well as downregulation of E-cadherin in AGS and HGC27 cells upon incubation with circ_0001789-exo (Fig. 2M). These data suggested that exosomal circ_0001789 mediated cell–cell communication to promote the progression of GC.

### Knockdown of circ_0001789 suppresses the malignancy of GC cells

**Fig. 3:**
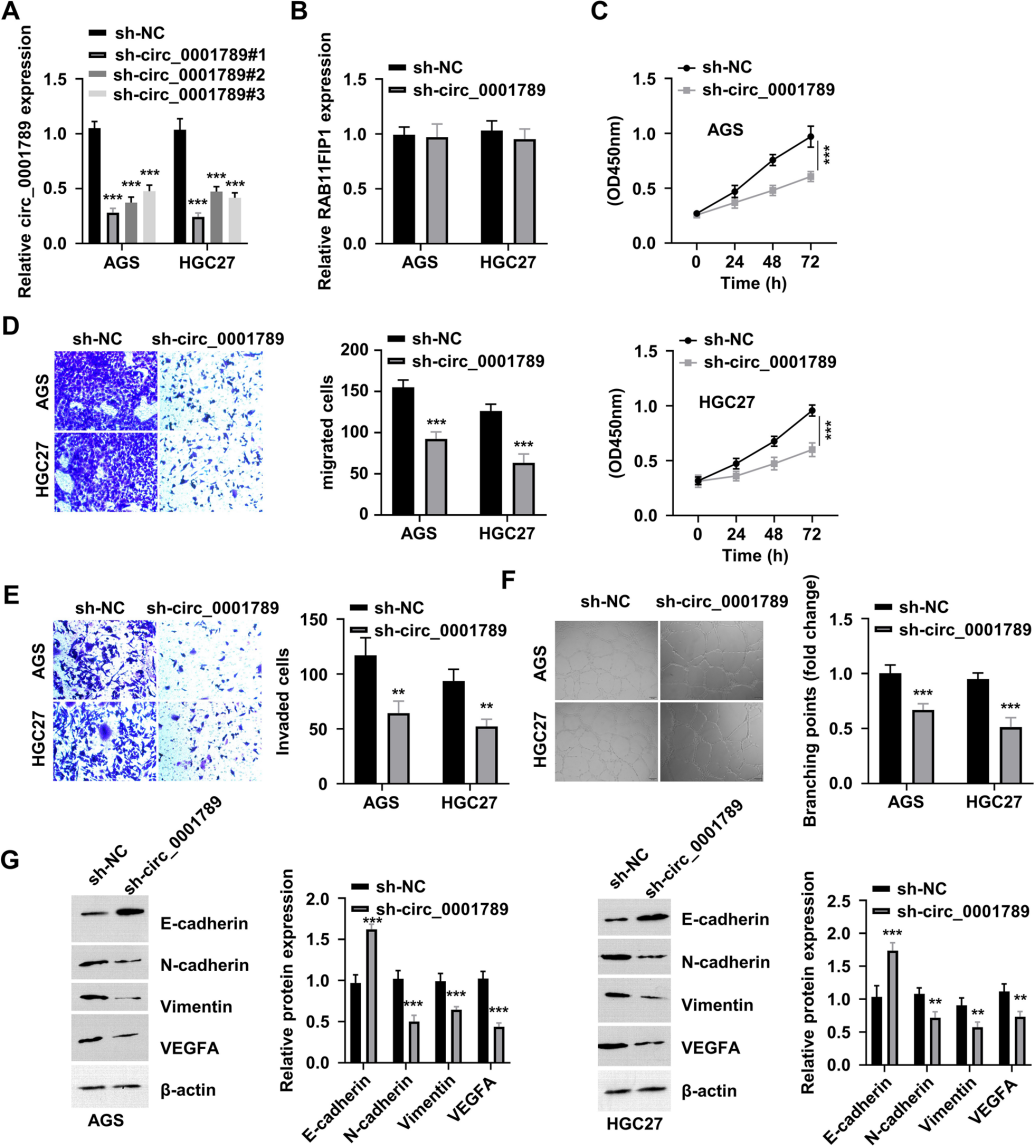
Knockdown of circ_0001789 impairs the malignant features of GC cells. (**A**) Two GC cell lines (AGS and HGC27) with high expression of circ_0001789 were selected for gene silencing using shRNAs targeting circ_0001789. **B** The RAB11 family interacting protein 1 mRNA levels in AGS and HGC27 cells transfected with sh-NC or sh-circ_0001789 were detected by reverse transcription-quantitative PCR. **C** Cell Counting Kit-8 proliferation assay in AGS and HGC27 cells transfected with sh-NC or sh-circ_0001789. **D** and **E** Cell migration and invasion assays in AGS and HGC27 cells transfected with sh-NC or sh-circ_0001789. **F** Tube formation assay in human umbilical vein endothelial cells incubated with exosome samples from AGS and HGC27 cells transfected with sh-NC or sh-circ_0001789. **G** Protein levels of E-cadherin, N-cadherin, vimentin and VEGF-A in AGS and HGC27 cells transfected with sh-NC or sh-circ_0001789. ^*^P < 0.05, ^**^P < 0.01, ^***^P < 0.001. circ, circular RNA; GC, gastric cancer; sh, small hairpin; NC, negative control

To verify the biological function of circ_0001789 in GC progression, circ_0001789 was silenced in two GC cell lines (AGS and HGC27) with high expression of circ_0001789 by using shRNAs. As depicted in Fig. 3A, the shRNAs targeting circ_0001789 (sh-circ_0001789#1, sh-circ_0001789#2 and sh-circ_0001789#3) significantly reduced circ_0001789 expression. Among them, sh-circ_0001789#1 showed the strongest knockdown effect and was therefore selected for subsequent experiments.

Notably, knockdown of circ_0001789 showed no effect on RAB11FIP1 mRNA expression (Fig. 3B). CCK-8 proliferation assay revealed that knockdown of circ_0001789 suppressed the proliferation of AGS and HGC27 cells (Fig. 3C). Silencing circ_0001789 also impaired the migration and invasion abilities of AGS and HGC27 cells (Fig. 3D, E). Exosomes from AGS and HGC27 cells transfected with sh-NC or sh-circ_0001789 were also isolated. Incubation of HUVECs with exosomes from sh-circ_0001789-treated cells led to impaired tube formation ability in comparison with that observed in cells treated with exosomes derived from control cells (Fig. 3F). Western blot results showed downregulation of N-cadherin, vimentin and VEGF-A, and upregulation of E-cadherin upon circ_0001789 silencing in AGS and HGC27 cells (Fig. 3G). Silencing experiments were also performed using sh-circ_0001789#2 (Additional file 1: Fig. S1), which showed consistent results with sh-circ_0001789#1. Together, these data showed that circ_0001789 was required to maintain the malignancy of GC cells.

### Circ_0001789 serves as a sponge of miR-140-3p

**Fig. 4:**
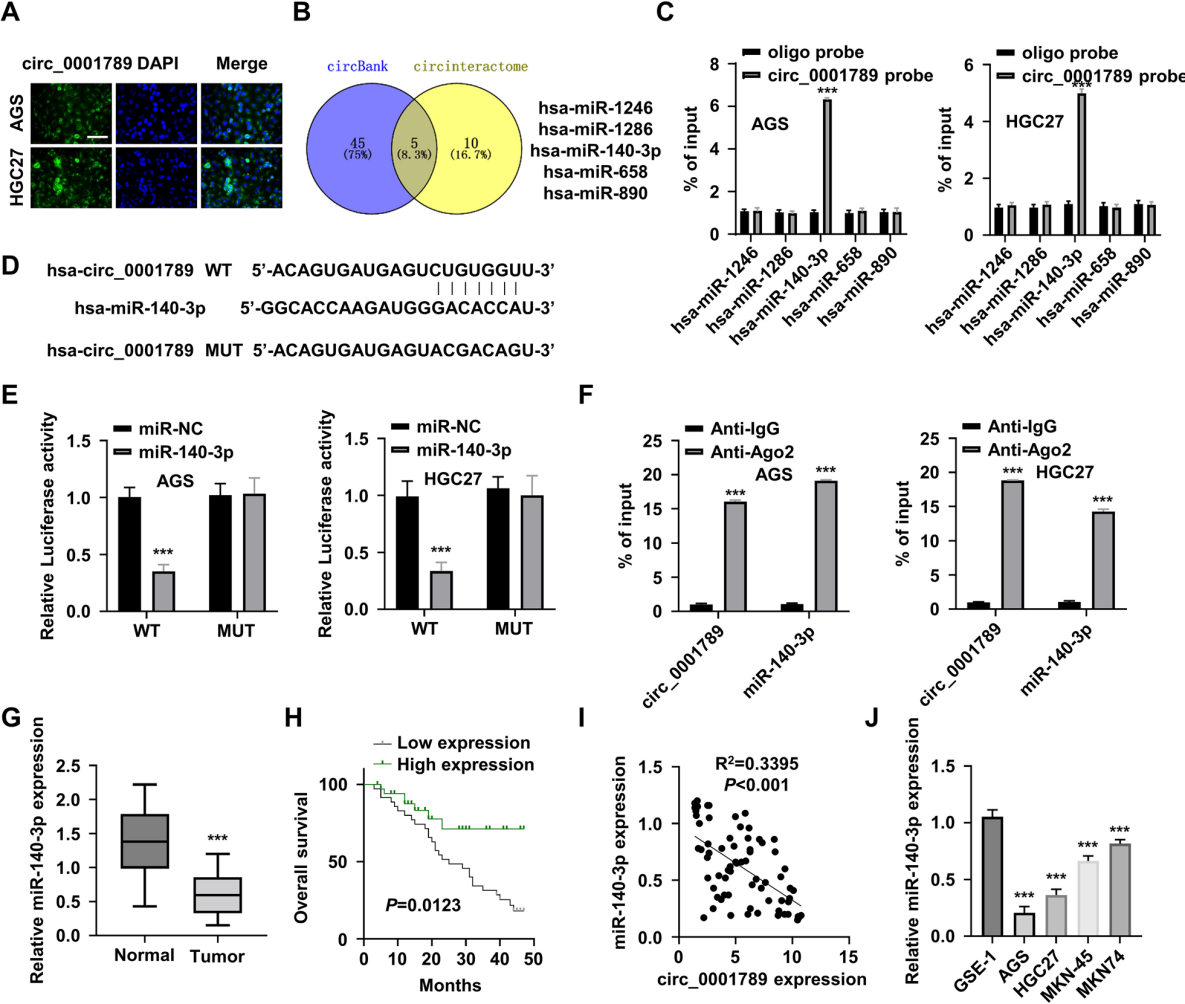
circ_0001789 targets miR-140-3p. **A** Fluorescence in situ hybridization images showing the subcellular localization of circ_0001789 in AGS and HGC27 cells. Green: circ_0001789; blue: Nucleus. Scale bar, 100 μm. **B** ‘circBank’ and ‘circInteractome’ database searching identified hsa-miR-1246, hsa-miR-1286, hsa-miR-140-3p, hsa-miR-658 and hsa-miR-890 as potential targets of circ_0001789. **C** RNA pull-down assay using biotin-labeled control oligo or circ_0001789 probe. RT-qPCR analysis showed significant enrichment of miR-140-3p by the circ_0001789 probe. **D** Schematics of the binding site of circ_0001789 sequence to miR-140-3p, and the corresponding mutations. **E** Dual luciferase reporter assay in AGS and HGC27 cells using reporter containing a wild-type or mutated binding site, in the presence of miR-negative control or miR-140-3p mimic. **F** RNA immunoprecipitation RT-qPCR analysis using anti-argonaute 2 or IgG isotype was performed in AGS and HGC27 cells. **G** RT-qPCR was used to detect the miR-140-3p expression level in normal gastric mucosa samples of healthy controls (n = 50) and in GC tissues (n = 70). **H** Kaplan–Meier plot showing the overall survival of patients with GC exhibiting high or low expression of miR-140-3p (n = 35 in each group). **I** Pearson’s correlation analysis showed a negative correlation between circ_0001789 and miR-140-3p in patients with GC (r > 0.6; P < 0.01). **J** RT-qPCR was used to detect the expression level of miR-140-3p in GC cell lines (AGS, HGC27, MKN-45 and MKN-74) and normal gastric epithelial cells (GES-1). ^*^P < 0.05, ^**^P < 0.01, ^***^P < 0.001. circ, circular RNA; miR, microRNA; hsa, *Homo sapiens*; GC, gastric cancer; RT-qPCR, reverse transcription-quantitative PCR

To verify the intracellular localization of circ_0001789 in AGS and HGC27 cells, FISH was performed using circ_0001789 as a probe. As shown in Fig. 4A, circ_0001789 was predominantly located in the cytoplasm of AGS and HGC27 cells. As circRNAs can act as molecular sponges of miRNAs [21], the ‘circBank’ and ‘circInteractome’ databases were employed to search for potential miRNA candidates of circ_0001789. A total of 5 miRNAs [*Homo sapiens* (hsa)-miR-1246, hsa-miR-1286, hsa-miR-140-3p, hsa-miR-658 and hsa-miR-890] were identified as potential targets of circ_0001789 (Fig. 4B). To validate the interactions, RNA pull-down assay was performed using a biotin-labeled circ_0001789 probe or a control oligo. The circ_0001789 probe significantly enriched miR-140-3p in comparison with the findings in the control oligo probe; however, this result was not observed for other miRNAs (Fig. 4C). To further confirm their interaction, dual luciferase reporter assay was performed using a reporter containing a WT or MUT binding site (Fig. 4D). The results showed that the presence miR-140-3p mimic significantly suppressed the luciferase activity of the circ_0001789 WT reporter, while there was no suppression in the MUT reporter activity (Fig. 4E). Furthermore, AGO2 RIP assay also demonstrated that both miR-140-3p and circ_0001789 were enriched in AGO2-containing complex (Fig. 4F). In comparison with its expression in normal gastric mucosa tissues, miR-140-3p was downregulated in GC tumor tissues (Fig. 4G), and patients with high expression of miR-140-3p showed a better overall survival compared with that of patients in the low-expression group (Fig. 4H). Pearson’s correlation coefficient analysis revealed a significant negative correlation between the expression of circ_0001789 and miR-140-3p in patients with GC (Fig. 4I). Furthermore, the RT-qPCR results revealed that miR-140-3p expression was reduced in GC cell lines (Fig. 4J). Together, these findings indicated that circ_0001789 could act as a sponge to negatively regulate miR-140-3p in GC cells.

### Inhibition of miR-140-3p partially rescues the effects of circ_0001789 silencing

**Fig. 5:**
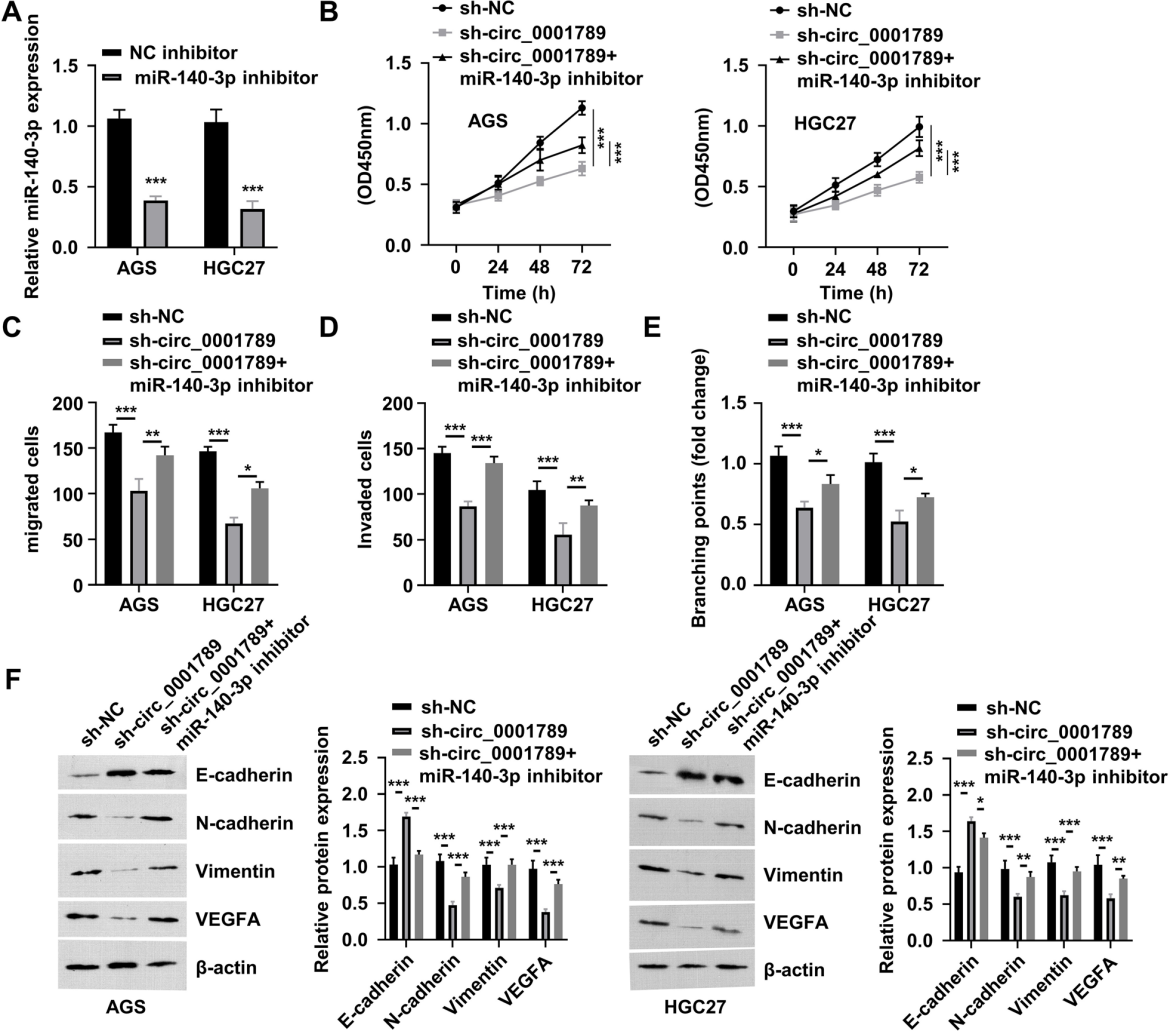
Knockdown of miR-140-3p partially reverses the inhibitory effect of circ_0001789 silencing. **A** Reverse transcription-quantitative PCR was used to detect the expression of miR-140-3p in AGS cells, while HGC27 cells were transfected with NC inhibitor or miR-140-3p inhibitor. **B** Cell Counting Kit-8 proliferation assay in AGS and HGC27 cells subjected to different treatments (sh-NC, sh-circ_0001789 or sh-circ_0001789 + miR-140-3p inhibitor). **C** and **D** Cell migration and invasion assays in AGS and HGC27 subjected to different treatments (sh-NC, sh-circ_0001789 or sh-circ_0001789 + miR-140-3p inhibitor). **E** Tube formation assay in human umbilical vein endothelial cells incubated with exosome samples from AGS and HGC27 cells exposed to different treatments (sh-NC, sh-circ_0001789 or sh-circ_0001789 + miR-140-3p inhibitor). **F** Protein levels of E-cadherin, N-cadherin, vimentin and VEGF-A in AGS and HGC27 cells subjected to different treatments (sh-NC, sh-circ_0001789 or sh-circ_0001789 + miR-140-3p inhibitor). ^*^P < 0.05, ^**^P < 0.01, ^***^P < 0.001. miR, microRNA; circ, circular RNA; sh, small hairpin; NC, negative control

To further investigate whether miR-140-3p mediates the downstream effects of circ_0001789, a miR-140-3p inhibitor was applied to suppress the expression of miR-140-3p (Fig. 5A). In the presence of a miR-140-3p inhibitor, the inhibitory effect of circ_0001789 silencing on the proliferation of AGS and HGC27 cells was attenuated (Fig. 5B). Inhibiting miR-140-3p also rescued the migratory and invasive abilities of GC cells upon circ_0001789 silencing (Fig. 5C, D), and the mir-140-3p inhibitor partially increased the tubulogenic ability of HUVECs following circ_0001789 silencing (Fig. 5E). Consistently, the protein levels of N-cadherin, vimentin and VEGF-A were largely rescued by the mir-140-3p inhibitor following circ_0001789 silencing, while the expression level of E-cadherin protein was suppressed by the mir-140-3p inhibitor (Fig. 5F). Therefore, these data suggested that miR-140-3p mediated the downstream effects of circ_0001789.

### Circ_0001789 sponges mir-140-3p to upregulate PAK2 expression

**Fig. 6:**
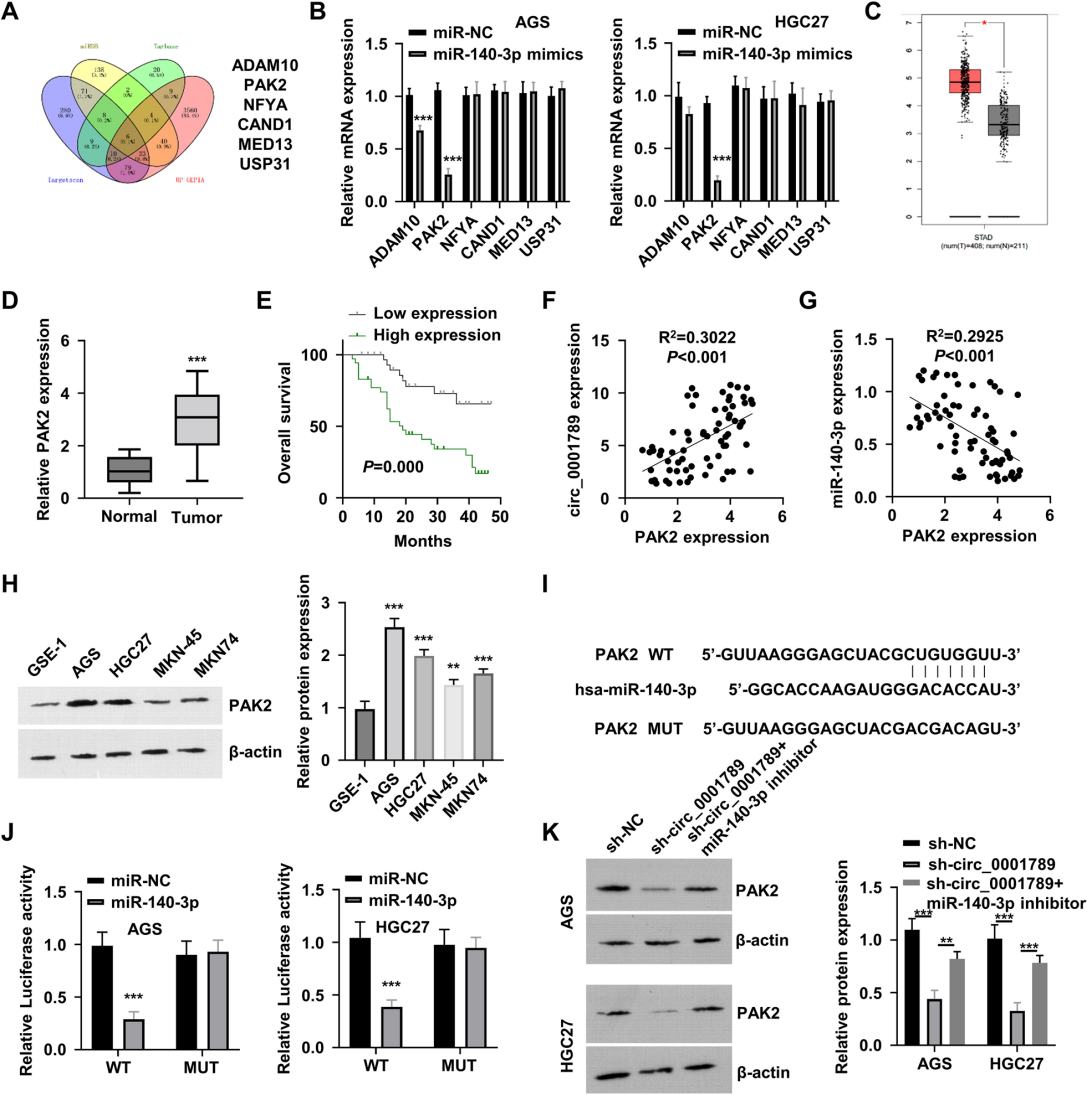
circ_0001789 sponges miR-140-3p to upregulate PAK2 expression. **A** The ‘Targetscan’, ‘miRDB’, ‘TarBase’ and ‘UP-GEPIA’ databases were employed to predict the downstream target mRNAs of miR-140-3p: ADAM10, PAK2, NFYA, CAND1, MED13 and USP31. **B** RT-qPCR was used to detect the candidate mRNA levels of ADAM10, PAK2, NFYA, CAND1, MED13 and USP31 in AGS and HGC27 cells transfected with miR-NC or miR-140-3p mimics. **C** The Cancer Genome Atlas database analysis of GC samples and normal gastric samples showed upregulation of PAK2 in GC. **D** RT-qPCR revealed the expression levels of PAK2 in normal gastric mucosa samples of healthy controls (n = 50) and GC tissues (n = 70). **E** Kaplan–Meier plot showing the overall survival of patients with GC exhibiting high or low expression of PAK2 (n = 35 in each group). **F** and **G** Pearson’s correlation analysis revealed a positive correlation between PAK2 and circ_0001789, and a negative correlation between PAK2 and miR-140-3p expression (r > 0.6, P < 0.01). **H** Western blot analysis of the PAK2 level in GC cell lines (AGS, HGC27, MKN-45 and MKN-74) and normal gastric epithelial cells (GES-1). **I** Schematic diagram of the binding site between miR-140-3p and the 3’ untranslated region of PAK2 mRNA, and the mutated binding sequence. **J** Dual luciferase reporter assay in AGS and HGC27 cells using a reporter containing a wild-type or a mutated binding site in the presence of miR-NC or miR-140-3p mimic. **K** PAK2 expression levels in different groups of AGS and HGC27 cells (sh-NC, sh- circ_0001789 or circ_0001789 + miR-140-3p inhibitor) were detected by western blotting. ^*^P < 0.05, ^**^P < 0.01, ^***^P < 0.001. circ, circular RNA; miR, microRNA; PAK2, p21 activated kinase 2; ADAM10, a disintegrin and metalloproteinase domain-containing protein 10; NFYA, nuclear transcription factor Y subunit α; CAND1, cullin associated and neddylation dissociated 1; MED13, mediator complex subunit 13; USP31, ubiquitin specific peptidase 31; RT-qPCR, reverse transcription-quantitative PCR; NC, negative control; GC, gastric cancer

To further explore the mRNA targets of miR-140-3p, the ‘TargetScan’, ‘miRDB’, ‘TarBase’ and ‘UP-GEPIA’ databases were employed to predict the downstream mRNA with a potential binding site for miR-140-3p. It was observed that the mRNAs of a disintegrin and metalloproteinase domain-containing protein 10 (ADAM10), PAK2, nuclear transcription factor Y subunit α (NFYA), cullin associated and neddylation dissociated 1 (CAND1), mediator complex subunit 13 and ubiquitin specific peptidase 31 (USP31) contained a potential miR-140-3p site (Fig. 6A). To validate this finding, GC cells were transfected with miR-NC and miR-140-3p mimics, and RT-qPCR was performed to detect the mRNA expression levels of these candidates in AGS and HGC27 cells. As shown in Fig. 6B, overexpression of miR-140-3p could downregulate the mRNA levels of ADAM10 and PAK2, with PAK2 mRNA showing the strongest downregulation. Furthermore, The Cancer Genome Atlas analysis between normal gastric and GC tissues showed that PAK2 was highly expressed in GC (Fig. 6C), which was further confirmed in clinical samples of normal gastric mucosa tissues and GC tumor samples by RT-qPCR (Fig. 6D).

High PAK2 expression was associated with poor prognosis in patients with GC (Fig. 6E). Pearson’s correlation analysis indicated that the expression levels of PAK2 and circ_0001789 were positively correlated (Fig. 6F), while the expression levels of PAK2 and miR-140-3p showed a negative correlation in the samples of patients with GC (Fig. 6G). Consistently, PAK2 was upregulated in GC cells in comparison with its expression in GES-1 cells (Fig. 6H). A luciferase reporter containing a WT or MUT binding site between PAK2 mRNA and miR-140-3p was also constructed, and dual luciferase reporter assay showed that overexpression of mir-140-3p could significantly inhibit the luciferase activity of the WT reporter, which was not observed in the MUT reporter (Fig. 6J). Knockdown of circ_0001789 also produced a negative effect on the expression level of PAK2 protein, which could be rescued by a miR-140-3p inhibitor (Fig. 6K).

### Overexpression of PAK2 reverses the inhibitory effect of miR-140-3p

**Fig. 7:**
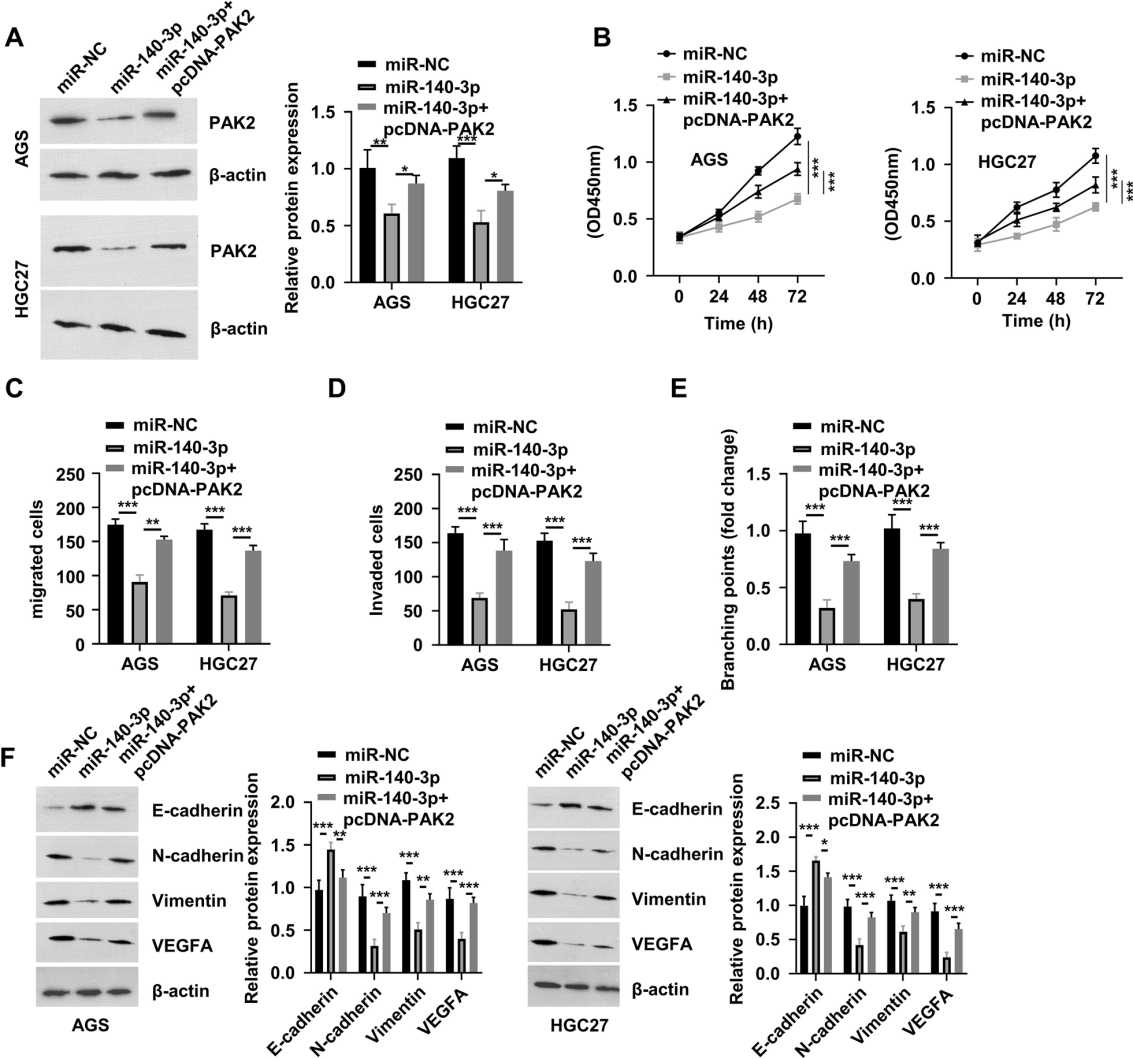
Overexpression of PAK2 reverses the inhibitory effect of miR-140-3p on gastric cancer. **A** Western blot analysis of PAK2 in AGS and HGC27 cells subjected to different treatments (miR-NC, miR-140-3p or miR-140-3p + pcDNA-PAK2). **B** Cell Counting Kit-8 assay was used to detect cell proliferation in AGS and HGC27 cells in different groups (miR-NC, miR-140-3p or miR-140-3p + pcDNA-PAK2). **C** and **D** Cell migration and invasion assays in AGS and HGC27 cells in different groups (miR-NC, miR-140-3p or miR-140-3p + pcDNA-PAK2). **E** Tube formation assay in human umbilical vein endothelial cells incubated with exosome samples from AGS and HGC27 cells subjected to different treatments (miR-NC, miR-140-3p or miR-140-3p + pcDNA-PAK2). **F** Protein levels of E-cadherin, N-cadherin, vimentin and VEGF-A in AGS and HGC27 cells subjected to different treatments (miR-NC, miR-140-3p or miR-140-3p + pcDNA-PAK2). ^*^P < 0.05, ^**^P < 0.01, ^***^P < 0.001. PAK2, p21 activated kinase 2; miR, microRNA; NC, negative control

To demonstrate that PAK2 mediates the effect of miR-140-3p, cells were transfected with miR-140-3p mimics that could downregulate PAK2 in GC cells (Fig. 7A), and co-transfection with the pcDNA-PAK2 vector increased PAK2 expression. Although overexpression of miR-140-3p inhibited cell proliferation, co-transfection with the pcDNA-PAK2 expression vector partially rescued cell proliferation in the presence of miR-140-3p mimics (Fig. 7B). Similarly, PAK2 overexpression also rescued the migratory and invasive abilities of GC cells following miR-140-3p overexpression (Fig. 7C, D), and PAK2 overexpression partially increased the tubulogenic ability of HUVECs upon miR-140-3p overexpression (Fig. 7E). Consistently, the protein levels of N-cadherin, vimentin and VEGF-A were reduced by miR-140-3p mimics, but largely rescued by PAK2 overexpression, while the expression level of E-cadherin protein was suppressed by PAK2 overexpression (Fig. 7F). Together, these data indicated that PAK2 mediated the functional role of miR-140-3p in controlling the malignancy of GC cells.

### *Knockdown of circ_0001789 inhibits tumorigenesis and metastasis of GC *in vivo

**Fig. 8:**
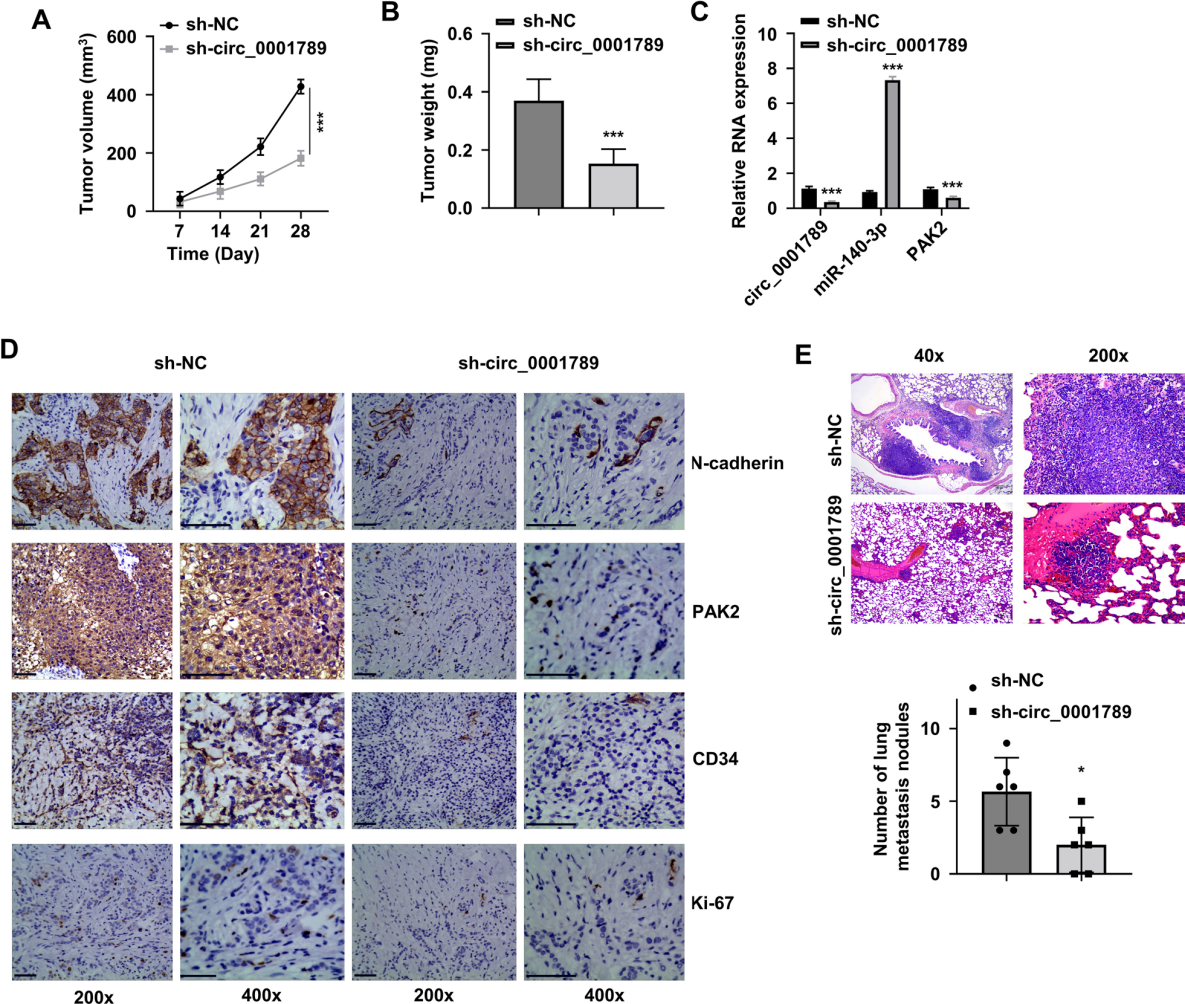
Silencing circ_0001789 suppresses the tumorigenesis and metastasis of GC cells in vivo. **A** Tumor volume of GC cells transfected with sh-NC and sh-circ_0001789 in nude mice. **B** Tumor weight of xenografts from nude mice was measured at the end of the experiment in the sh-NC and sh-circ_0001789 groups. **C** Reverse transcription-quantitative PCR analysis of the expression level of circ_0001789, microRNA-140-3p and PAK2 in the xenografts of nude mice. **D** Immunohistochemistry was performed to detect the expression of Ki-67, PAK2, N-cadherin and CD34 in the xenografts of nude mice in the sh-NC and sh-circ_0001789 groups. **E** Hematoxylin and eosin staining of lung metastatic nodules in nude mice of the sh-NC and sh-circ_0001789 groups. ^*^P < 0.05, ^**^P < 0.01, ^***^P < 0.001. circ, circular RNA; GC, gastric cancer; NC, negative control; sh, small hairpin; PAK2, p21 activated kinase 2

To further validate the oncogenic role of circ_0001789 in vivo, AGS cells transfected with sh-NC and sh-circ-0001789 were injected subcutaneously into nude mice. The tumor growth curve showed that, compared with the effects of cells transfected with sh-NC, circ_0001789 silencing in the sh-circ_0001789 group attenuated the increase in tumor volume and reduced the tumor weight in vivo (Fig. 8A, B). Consistent with the in vitro data, silencing circ_0001789 led to the upregulation of mir-140-3p and the downregulation of PAK2 in the isolated xenograft samples (Fig. 8C).

IHC staining of different markers was performed, which showed that the expression levels of Ki-67, PAK2, CD34 and N-cadherin were significantly reduced in the silencing group compared with those in the sh-NC group (Fig. 8D). In addition, H&E staining was performed in lung tissues to examine metastasis. In the group of GC tumor with circ_0001789 silencing, the number of observed metastatic nodules in lung tissues was lower than that in the sh-NC group (Fig. 8E). These data supported that circ_0001789 was an oncogenic factor supporting the tumorigenesis and metastasis of GC in vivo*.*

## Discussion

The increasing incidence of GC in the world imposes a burden on public health [26]. The 5-year survival rate of GC remains  < 30% [27], which is mainly due to the lack of efficient early diagnostic markers and the compromised treatment outcome in patients with late-stage GC [28, 29]. Recurrence and metastasis frequently occur in patients with GC who undergo treatments such as radical gastrectomy and chemotherapy [29]. Previous studies also elucidated the roles of other non-coding RNA such as miRNAs in the development of GC [30, 31]. However, the functional role of circRNAs and their underlying mechanisms in GC have yet to be explored. The present study employed a published dataset profiling differentially expressed circRNAs between GC tissues and normal gastric mucosa tissues, which revealed the upregulation of hsa_circ_0001789 (host gene symbol Rab11fip1, length 443 kb) in GC tumors. This observation was also confirmed in gastric mucosa tissues collected from healthy controls and patients with GC. Notably, the expression level of circ_0001789 in patients with metastatic tumors was much higher than that in the group without metastasis, and patients with high circ_0001789 expression showed worse overall survival compared with that shown by patients with lower expression of circ_0001789. These results indicated that circ_0001789 may serve as a biomarker of poor prognosis.

Since circ_0001789 is derived from the exon2 of RAB11FIP1, its circular structure was confirmed by comparing its stability with RAB11FIP1 mRNA in AGS and HGC27 cells following actinomycin D and RNase R treatment. To further confirm its functional role, loss-of-function analysis was performed by shRNA-mediated silencing, which revealed that circ_0001789 was indispensable for the malignant phenotype of GC cells, including their proliferation, migration and invasion, as well as the EMT process. The oncogenic role of circ_0001789 in tumorigenesis and metastasis was further validated in nude mice. Together, the present data supported the notion that circ_0001789 was upregulated in GC cells to promote the progression of GC.

Accumulating evidence suggests that exosomes derived from cancer cells played an important function in cancer biology [9]. Exosomal circRNA could be a biomarker in liquid biopsy for early diagnosis in patients with cancer [32, 33]. The present study further demonstrated that circ_0001789 in exosomes derived from GC cells not only promoted the malignancy of GC cells receiving exosomes, but also augmented the angiogenic potential of endothelial cells. These data indicated that exosomal circ_0001789 could facilitate the malignant progression of GC tumor tissues by regulating the vascularization and EMT, which warrants further investigation in a mouse model.

To explore the network of circ_0001789, the current study further demonstrated that miR-430-3p could be sponged and negatively regulated by circ_0001789. In contrast to the oncogenic function of circ_0001789, overexpression of miR-430-3p suppressed cell proliferation, migration and invasion, as well as EMT markers. Inhibition of miR-430-3p also rescued the effects of circ_0001789 silencing. These data were consistent with previous findings that miR-140-3p served as a tumor suppressor to inhibit the metastasis of various cancer types [13, 14].

PAK2, a promising anticancer target in GC cells [16–18], was shown to be a downstream target of miR-430-3p. Its expression was negatively correlated with miR-430-3p, but positively correlated with circ_0001789 in patients with GC, indicating that it is regulated by the circ_0001789/miR-430-3p axis. miR-430-3p overexpression or circ_0001789 silencing reduced PAK2 expression. Importantly, overexpression of PAK2 rescued the inhibitory effects of miR-430-3p overexpression or circ_0001789 silencing on cell proliferation, migration and invasion, and EMT. Consistently, PAK2 has been shown to act as an effector to confer drug resistance and malignant properties in breast and cervical cancer [34, 35]. Therefore, these data suggested that PAK2 was a downstream mediator to support the oncogenic effects of circ_0001789 in GC cells.

Several questions remains to be investigated by the future studies. First, what are the mechanisms underlying circ_0001789 overexpression in GC? The answer to this question is the key to the formulation of intervention strategies. Further, how PAK2 mediates the malignant phenotype in GC needs further clarification. Perhaps the exploration of the roles of circ_0001789/miR-140-3p/PAK2 axis in important cellular organelle such as mitochondria would provide novel insights. Since circRNAs are promising candidates for diagnosis and therapy, the application of sensitive nanoparticle technology could be considered for in vivo diagnosis and drug delivery [36]. Besides, a multi-center prospective study involving a large cohort of patients is needed to further validate the prognostic value of circ_0001789 for the progression of GC [37].

In summary, the present study identified a novel circRNA, circ_0001789, which was overexpressed in GC tissue samples and cell lines to confer malignant phenotypes. The present data suggest that upregulation of circ_0001789 is associated with malignant progression of GC and poor prognosis in GC patients, and that miR-140-3p/PAK2 serves as the downstream axis to mediate the oncogenic effect of circ_0001789. Besides, silencing circ_0001789 suppressed the tumorigenesis of GC cell in the animal model. Overall, our data identified a novel regulatory module in dictating the malignant progression of GC, indicating that targeting circ_0001789/miR-140-3p/PAK2 axis could serve a novel strategy for interventional management of GC.

## Supplementary Information

**Additional file 1: Figure S1.** (A) Two gastric cancer cell lines (AGS and HGC27) with high expression of circ_0001789 were selected for gene silencing using sh-RNA targeting circ_0001789 (sh- circ_0001789#2). (B) Cell Counting Kit-8 proliferation assay in AGS and HGC27 cells transfected with sh-NC or sh-circ_0001789#2. (C and D) Cell migration and invasion assays in AGS and HGC27 cells transfected with sh-NC or sh-circ_0001789#2. (E) Tube formation assay in human umbilical vein endothelial cells incubated with exosome samples from AGS and HGC27 cells transfected with sh-NC or sh-circ_0001789#2. (F) Protein levels of E-cadherin, N-cadherin, vimentin and VEGF-A in AGS and HGC27 cells transfected with sh-NC or sh-circ_0001789#2. ^*^P<0.05, ^**^P<0.01, ^***^P<0.001. circ, circular RNA; sh, small hairpin; NC, negative control.

**Additional file 2: Table S2.** Primer sequences for qRT-PCR.

**Additional file 3: Table S1.** shRNA sequence.

## Data Availability

The data generated in the present study is available from the corresponding author upon reasonable request.
