# Supplementary figures and images for: Circular RNA 0001789 sponges miR-140-3p and regulates PAK2 to promote the progression of gastric cancer
Source: J Transl Med. 2023 Feb 5;21:83. doi: 10.1186/s12967-022-03853-2 (PMC9901162; doi:10.1186/s12967-022-03853-2)

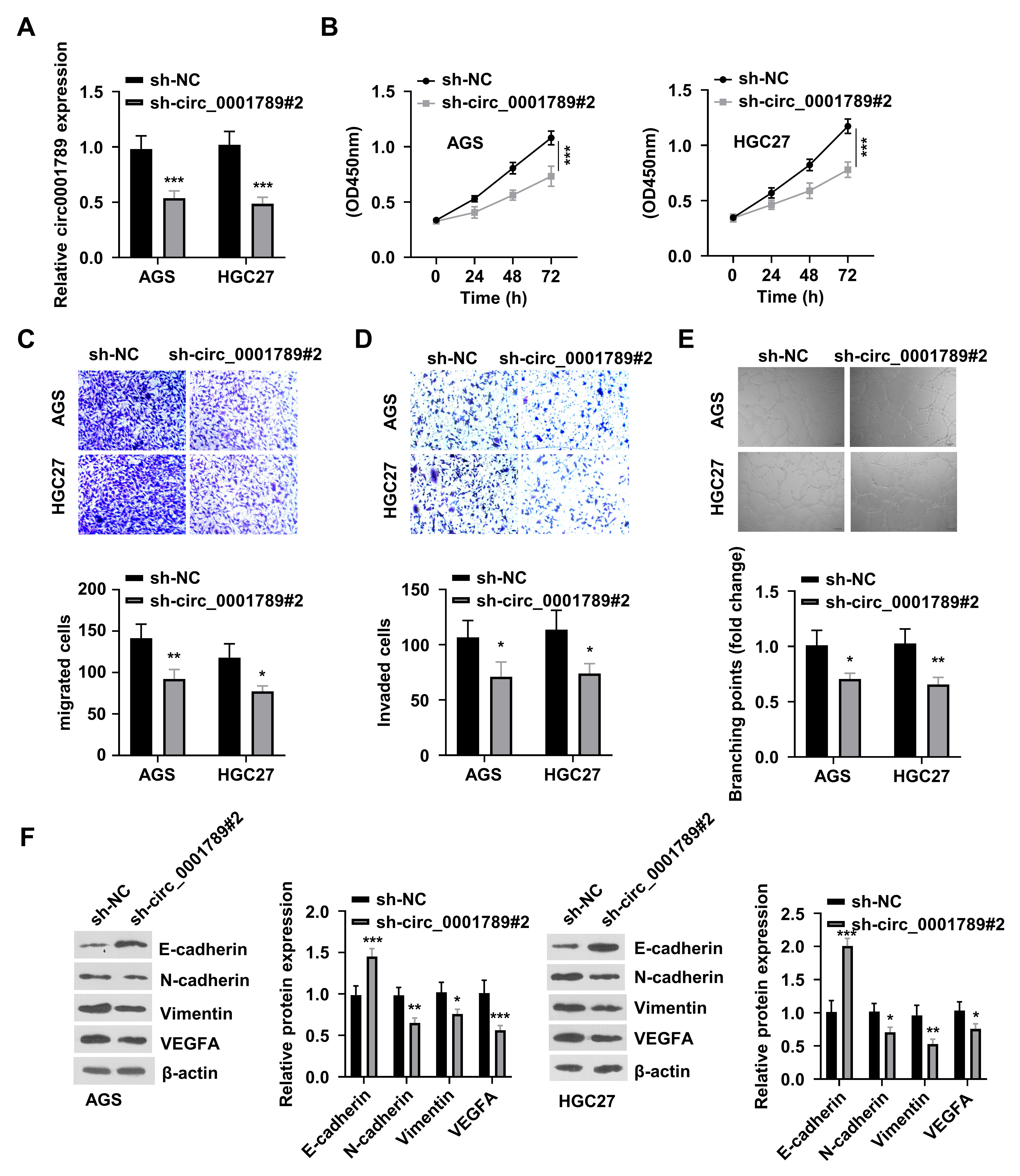

Supplement: Supplementary file 1 — Additional file 1: Figure S1. (A) Two gastric cancer cell lines (AGS and HGC27) with high expression of circ_0001789 were selected for gene silencing using sh-RNA targeting circ_0001789 (sh- circ_0001789#2). (B) Cell Counting Kit-8 proliferation assay in AGS and HGC27 cells transfected with sh-NC or sh-circ_0001789#2. (C and D) Cell migration and invasion assays in AGS and HGC27 cells transfected with sh-NC or sh-circ_0001789#2. (E) Tube formation assay in human umbilical vein endothelial cells incubated with exosome samples from AGS and HGC27 cells transfected with sh-NC or sh-circ_0001789#2. (F) Protein levels of E-cadherin, N-cadherin, vimentin and VEGF-A in AGS and HGC27 cells transfected with sh-NC or sh-circ_0001789#2. *P<0.05, **P<0.01, ***P<0.001. circ, circular RNA; sh, small hairpin; NC, negative control. [file 12967_2022_3853_MOESM1_ESM.jpg]
