# Supplementary material for: Circular RNA 0001789 sponges miR-140-3p and regulates PAK2 to promote the progression of gastric cancer
Source: J Transl Med. 2023 Feb 5;21:83. doi: 10.1186/s12967-022-03853-2 (PMC9901162; doi:10.1186/s12967-022-03853-2)
Supplement: Supplementary file 2 — Additional file 2: Table S2. Primer sequences for qRT-PCR. [file 12967_2022_3853_MOESM2_ESM.docx]

| shRNA | sequence |
| --- | --- |
| Sh-NC | CCTAAGGTTAAGTCGCCCTCGCTCACCGAGCGAGGGCGACTTAACCTTAGG |
| Sh- circ_0001789#1 | TCTCGGAATCCATTTGGAAAGCTGAATCACCGATTCAGCTTTCCAAATGGATTCCGAGA |
| Sh- circ_0001789#2 | GCCATCATCCCTAGCACGACACCTTCACCGAAGGTGTCGTGCTAGGGATGATGGC |
| Sh- circ_0001789#3 | CGGTCGACAGTGATGATGAGTCTGTCACCGACAGACTCATCATCACTGTCGACCG |

Table S1 shRNA sequence
