# Supplementary material for: Circular RNA 0001789 sponges miR-140-3p and regulates PAK2 to promote the progression of gastric cancer
Source: J Transl Med. 2023 Feb 5;21:83. doi: 10.1186/s12967-022-03853-2 (PMC9901162; doi:10.1186/s12967-022-03853-2)
Supplement: Supplementary file 3 — Additional file 3: Table S1. shRNA sequence. [file 12967_2022_3853_MOESM3_ESM.docx]

| Primer name | Forward (3’-5’) | Reverse (3’-5’) |
| --- | --- | --- |
| Actin | CATGTACGTTGCTATCCAGGC | CTCCTTAATGTCACGCACGAT |
| U6 | CACGAATTTGCGTGTCATCCTT | GTGTAA CACGTCTATACGCCCA |
| RAB11FIP1 | GCTCGGCCTCGACAAGTTC | ACTTATACCACTGCGTCTTCCT |
| miR-140-3p | TGCGGCAGTGGTTTTACCCTATG | CCAGTGCAGGGTCCGAGGT |
| PAK2 | TGAGCACACCATCCATGTTGG | AGGTCTGTAGTAATCGAGCCC |
| circ_0001789 | CTCGCTACTCTACCTGCTGGA | CAGACGACATGTGCGAGCTA |
| miR-1246 | CTCAACTGGTGTCGTGGAG | TGGCCTGTCTCAACTGAATTG |
| miR-1286 | GGGTTTGTTCGTTCGGCTC | TGGTGTCGTGGAGTCG CTCA |
| miR-658 | CTCCAGCTGGGAATATAACAC | TGGTGTCGTGGAGTCG GCAAT |
| miR-890 | CTCAACTGGTGTCGTGGAGTC | ACTGGAAGCTCAACTGAATTGC |
| ADAM10 | ATGGGAGGTCAGTATGGGAATC | ACTGCTCTTTTGGCACGCT |
| NFYA | CAGTGGAGGCCAGCTAATCAC | CCAGGTGGGACCAACTGTATT |
| CAND1 | AGCGCCTCGTACCACATTTC | TGCAGTTCCGTCATCAAATCA |
| MED13 | GGGTGAAGACCCCAGTTTTG | GGAAAGTCCATTCTCCCACACT |
| USP31 | CTGTGGCTTTTGGACCGAGTT | CCTCAGGCATCATATCAGTCTCT |

Table S2. Primer sequences for qRT-PCR
